# Tocopheryl quinone improves non-alcoholic steatohepatitis (NASH) associated dysmetabolism of glucose and lipids by upregulating the expression of glucagon-like peptide 1 (GLP-1) *via* restoring the balance of intestinal flora in rats 

**DOI:** 10.1080/13880209.2021.1916542

**Published:** 2021-06-17

**Authors:** Tao Sun, Bing Zhang, Qing-jing Ru, Xiao-mei Chen, Bo-dong Lv

**Affiliations:** a The Second Clinical Medical College of Zhejiang Chinese Medical University, Hangzhou, PR China; b Department of Hepatology, The Second Affiliated Hospital of Zhejiang Chinese Medical University, Hangzhou, PR China; c Department of Traditional Chinese Medicine, Hangzhou First People’s Hospital, Zhejiang University School of Medicine, Hangzhou, PR China

**Keywords:** Diabetes mellitus, high cholesterol, cholate diet

## Abstract

**Context:**

Glucagon-like peptide 1 (GLP-1) and α-tocopheryl quinone can promote the growth of intestinal flora and affect the pathogenesis of non-alcoholic steatohepatitis (NASH).

**Objective:**

This study determines the molecular mechanism of the effect of tocopheryl quinone in the treatment of high cholesterol and cholate diet (HFCC)-induced NASH.

**Materials and methods:**

Thirty-two male Sprague Dawley (SD) rats grouped as lean control (LC), LC + tocopheryl quinone (1 mL of 3 × 10^6^ dpm tocopheryl quinone *via* i.p. injection), HFCC (5.1 kcal/g of fat diet), and HFCC + tocopheryl quinone. Profiles of intestinal flora were assessed by 16S ribosomal ribonucleic acid–based analysis. Levels and activity of GLP-1, interleukin 6 (IL-6) and tumour necrosis factor alpha (TNF-α) in intestinal tissues were detected by immunohistochemistry (IHC), Western blot and enzyme-linked immunosorbent assay (ELISA).

**Results:**

HFCC rats presented higher levels of cholesterol, low-density lipoprotein (LDL) and high-density lipoprotein (HDL), while tocopheryl quinone reversed the effects of HFCC. HFCC dysregulated malondialdehyde (MDA), glutathione (GSH), superoxide dismutase (SOD), Vitamin E, 12-hydroxyeicosatetraenoic acid (12-HETE), 13-hydroxyoctadecadienoic acid (13-HODE) and nuclear factor kappa B (NF-κB), and the effects of HFCC were reversed by the treatment of tocopheryl quinone. Also, GLP-1 in the HFCC group was down-regulated while the IL-6 and TNF-α activity and endotoxins were all up-regulated. HFCC significantly decreased the number and diversity of bacteria, whereas tocopheryl quinone substantially restored the balance of intestinal flora and promoted the growth of both *Bacteroides* and *Lactobacilli in vitro*.

**Discussion and conclusions:**

α-Tocopheryl quinone relieves HFCC-induced NASH *via* regulating oxidative stress, GLP-1 expression, intestinal flora imbalance, and the metabolism of glucose and lipids.

## Introduction

The diagnosis of non-alcoholic fatty liver disease (NAFLD) is supported by the imaging or histology evidence of hepatic steatosis, and no other causes for secondary hepatic fat accumulation, i.e., significant alcohol consumption, use of steatogenic medication or hereditary disorders, shall be presented. Furthermore, NAFLD can be divided into two distinct pathological conditions, i.e., non-alcoholic steatohepatitis (NASH) and non-alcoholic fatty liver (NAFL) (Shukla et al. 2011). In addition, liver fibrosis patients often suffer from diarrhoea, abdominal distension and other symptoms in the gastrointestinal tract that may be caused by the imbalance of flora in the intestine (Bauer et al. 2000). As a result, the flora in the intestine is speculated to play an essential role in the pathogenesis of NASH and NAFLD. Accumulating data indicated that the gut microbiota can transmit signals through the intestine to the liver to play an important role in lipotoxicity (Lakhani et al. 2008; Madrid et al. 2011).

Tocopheryl quinone (TQ) was shown to possess unique physiochemical properties and is involved in a wide range of biomolecular processes (Cornwell and Ma 2007). In addition, TQ was found to be a dramatically more potent inhibitor of ferroptosis (Dolfi et al. 2013). Also, TQ and all other products of vitamin E metabolism have been reported to be potent modulators of 5-lipoxygenase (5-LOX), 15-lipoxygenase (15-LOX) (Dolfi et al. 2013) and nuclear receptors like PXR. Moreover, the gut microbiota has been reported to metabolize vitamin E, and the bioavailability of vitamin E increased by antibiotics is due to its decreased degradation by gut microbes.

As a type of polypeptides mainly produced by L cells in the ileum, GLP‐1 is used in clinical applications to treat type II diabetes mellitus (DM) associated with obesity (Prasad-Reddy and Isaacs 2015). The role of GLP‐1 in promoting weight loss is manifested in multiple clinical trials (Monami et al. 2012; Blackman et al. 2016). GLP‐1 plays an important role in keeping the homeostasis of glucose in the body while maintaining the equilibrium of flora population in the intestine (Farilla et al. 2002; Inoue et al. 2015). Using an animal model free of germs, it was demonstrated that the microbial ecosystems in the gut play an essential role in keeping GLP‐1 sensitivity, therefore, suggesting a new way to increase the effectiveness of antidiabetic drugs by utilizing the role of GLP‐1 in glucose homeostasis regulation (Fenn et al. 2017; Yuan et al. 2018).

TQ has been reported to influence the balance of gut microbiota, and the imbalance of gut microbiota identified in NASH and NAFLD was found to play a role in the pathogenesis of DM. In this study, we hypothesized that α-tocopheryl quinone would potentially influence the metabolism of glucose or lipid to improve NASH-associated dysmetabolism of glucose or lipids. Therefore, we treated NASH rats with α-tocopheryl quinone and studied its effect on the metabolism of glucose and lipids, the level of oxidative stress, intestinal flora imbalance, and the level of Glucagon-like peptide 1 (GLP-1) expression.

## Materials and methods

### Animal model and grouping

A total of 32 male Sprague Dawley (SD) rats (6 weeks old, weighing 163–188 g) were purchased from Institutional animal center and used as the experimental animals of this study. These rats were divided into four groups (*n* = 8): lean control (LC) group (used as a control group, in which the rats were given 0.5% sodium carboxymethyl cellulose in saline *via* gavage); LC + tocopheryl quinone group (used as another control group, in which the rats were given 0.5% sodium carboxymethyl cellulose in saline *via* gavage + 3 × 10^6^ dpm of tocopheryl quinone *via* intraperitoneal injection); high cholesterol and cholate diet (HFCC) group; HFCC + tocopheryl quinone group (the rats were given 20 mg/kg of tocopheryl quinone *via* intraperitoneal injection). To establish a diabetic model, the level of cholesterol in each group was measured once every 2 weeks. Since the treatment with tocopheryl quinone is known to decrease the levels of cholesterol in rats fed with HFCC, the low-density lipoprotein (LDL) level in various groups was also examined once every 2 weeks. Furthermore, serum and intestinal tissues were collected from all animals to measure their levels of redox indicators, such as malondialdehyde (MDA), glutathione (GSH), SOD and vitamin E. And the blood samples from all rats were collected for oral glucose tolerance test (OGTT). Each animal operation procedure was carried out in strict compliance with national laws and international guidelines for Animal Welfare. The Ethics Committee of The Second Clinical Medical College of Zhejiang Chinese Medicine University have approved the protocols of this study (Approval ID: 2018-KL-005-01).

### Animal treatment

Subsequently, to inspect the effects of tocopheryl quinone on the proliferation of bacteria in the intestine of rats, 20 mg/kg of tocopheryl quinone were administered intraperitoneally in 1 mL of liquid. During the experiment, the SD rats were housed in type IV standard cages fitted with a standard nest material, with two rats in each cage. And the animal facility was maintained at 22 ± 1 °C and 60% ± 5% humidity, with a 12 h light/dark cycle. The rats in the LC group were given free access to a standard chow and unlimited water. The rats in the HFCC group were given 10 weeks of a high-fat diet containing 5.1 kcal/g of fat. The food uptake and the weight of all rats were monitored daily during the study, along with their serum levels of alanine transaminase, lipoprotein, aspartate transaminase, cholesterol and triglycerides. In addition, a test of oral glucose tolerance (intragastric gavage, 2 g/kg body weight; 200 g/L solution) was carried out once a week after 10 weeks of the experiment by collecting tail vein blood samples at 0, 2, 4, 6, 8, 10 and 12 week after the initiation of a glucose challenge.

### H&E staining

The collected liver tissue samples were fixed using 10% formalin, embedded in paraffin and sliced into 2.5 μm sections for subsequent staining with H&E following casual routine.

### Liquid chromatography online electrospray ionization tandem mass spectrometry

Levels of vitamin E, 12-hydroxyeicosatetraenoic acid (12-HETE) and 13-hydroxyoctadecadienoic acid (13-HODE) were quantified using liquid chromatography online electrospray ionization tandem mass spectrometry (Liquid Chromatography Electrospray Ionization Tandem Mass Spectrometric) following routine procedures.

### Measurement of biochemical indicators

The profiles of serum biochemical indicators, including serum levels of alanine transaminase, lipoprotein, aspartate transaminase, cholesterol and triglycerides, were measured using a TBA-40FR Biochemistry Auto-analyzer (Toshiba, Tokyo, Japan).

### Evaluation of oxidative stress

Blood samples were collected from the heart of rats into EDTA-containing blood collection tubes. In addition, intestinal tissue samples were collected from the rats and homogenized. Subsequently, the supernatant of all samples was collected *via* 20 min of centrifugation at 1500 *g*, and the concentrations of antioxidant enzymes in the supernatant were determined with a superoxide dismutase (SOD) assay kit (Jiancheng Bioengineering, Nanjing, China). In addition, the concentrations of MDA, GSH and vitamin E in the supernatant were also evaluated using respectively assay kits (Jiancheng Bioengineering, Nanjing, China) based on the kit instructions.

### Colony enumeration

To assay the effects of 10 μmol/dm^3^ LG treatment on the proliferation of *Lactobacillus acidophilus* and *Bacteroides thetaiotaomicron*, the microbiota in rat gut was collected in the early stage of DM induction by mixing LG with a regular medium prior to 37 °C culture under anaerobic conditions. After the 600 nm optical density in bacterial broth was ≥ 1, the rate of bacterial proliferation was assayed with a spectrophotometer (GE Healthcare, Pittsburgh, PA).

### Analysis of 16S ribosomal RNA

Subsequently, the profiles of intestinal flora in the rats were assessed by an analysis based on 16S ribosomal RNA. In brief, the genomic DNA collected from the rat faecal samples was extracted with a TIANamp Stool DNA Assay Kit (Tiangen, Beijing, China). And the content of 16S ribosomal RNA was determined using PCR denaturing gradient gel electrophoresis in conjunction with a reverse primer of 5′‐GTA TTA CCG CGG CTG CTG GCA C‐3′ and a forward primer of 5′‐CGC CCG GGG CGC GCC CCG GGC GGG GCG GGG GCA CGG GGG GAC TCC TAC GGG AGG CAG CAG T‐3′ to evaluate the growth profiles of the bacterial ecosystem.

### IHC assay

The levels of GLP-1 in intestinal tissues were measured by IHC assay. In brief, intestinal tissue samples were collected from the rats, fixed in a pH 7.4 solution of 10% formalin, paraffin embedded, and sliced into 2 µm thick sections. After de-paraffinization with xylene, the sections were blocked for 15 min by 0.3% hydrogen peroxide, incubated at 100 °C for 30 min with a pH 6.0 buffer of 0.01 mol/L citrate to retrieve antigens, and further blocked with 10% foetal bovine serum. Then, the sections were stained with anti-GLP-1 and anti-nuclear factor-kappa B (anti-NF-κB) primary antibodies for 24 h at 4 °C (Cell Signalling Technology, Denver, CA), followed by 1 h of incubation with secondary antibodies and counter-staining with a DAB substrate (Sigma-Aldrich, St. Louis, MO) at room temperature. Subsequently, the positive expression of GLP-1 or NF-κB was assessed under a BH-2 Olympus light microscope (Olympus, Tokyo, Japan).

### Western blot analysis

The protein expression of GLP-1 in the intestinal tissue samples collected from the rat groups was measured using a routine Western blot assay. In brief, the next step, the collected protein content by centrifugation was loaded onto a 10% SDS-PAGE gel and then blotted onto a nitrocellulose membrane, and was then incubated against primary anti-GLP-1 antibodies (Santa Cruz Biotechnology, Santa Cruz, CA) and corresponding HRP labelled secondary antibodies (Santa Cruz Biotechnology, Santa Cruz, CA).

### ELISA assay

The activity of plasma GLP-1 in collected blood samples was measured using a commercial ELISA assay kit based on the instructions provided by the manufacturer (NeoBioscience, Beijing, China). Intestinal tissue samples collected from the animal groups were utilized to measure the activity of IL-6 and TNF-α based on the instructions provided by the manufacturer (NeoBioscience, Beijing, China).

### Statistical analysis

The statistical analysis was carried out with SPSS version 21.0 (IBM, Armonk, NY). The differences between two groups were compared by Student’s *t*-test while differences among different groups were analysed with one-way analysis of variance (ANOVA) with Bonferroni correction being utilized for *post hoc* corrections. The *p* value of significance was set to 0.05.

## Results

### Effects of HFCC and HFCC+ tocopheryl quinone on cholesterol levels in rats

**Figure 1.**
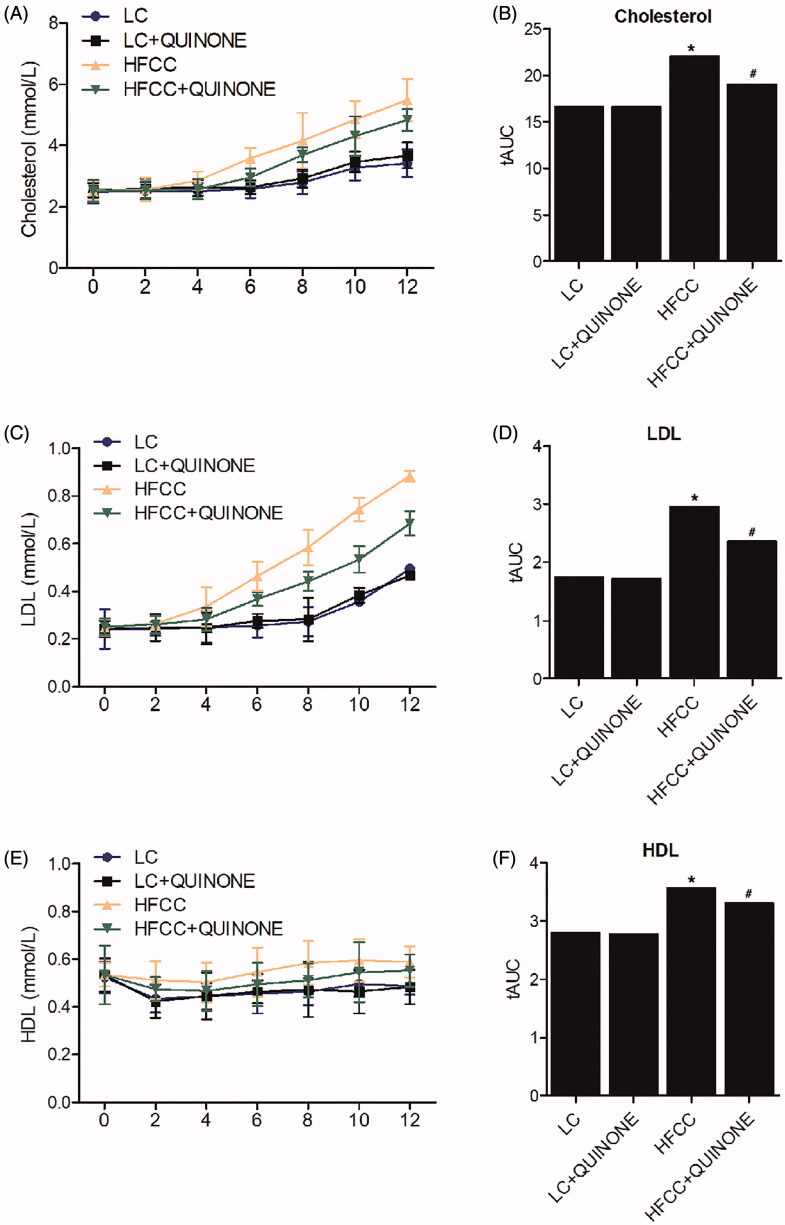
Cholesterol, LDL and HDL levels at different time points in LC group, LC + tocopheryl quinone group, HFCC group and HFCC + tocopheryl quinone group. The treatment with HFCC and tocopheryl quinone increased and decreased the levels of cholesterol, LDL and HDL, respectively (*n* = 3; *p value < 0.05 *vs.* LC group; ^#^*p* value < 0.05 *vs.* HFCC group). (A) Cholesterol levels in the four groups in weeks 0, 2, 4, 6, 8, 10 and 12; (B) Area under the curve of cholesterol levels in the four groups; (C) LDL levels in the four groups in weeks 0, 2, 4, 6, 8, 10 and 12; (D) Area under the curve of LDL levels in the four groups; (E) HDL levels in the four groups in weeks 0, 2, 4, 6, 8, 10 and 12; (F) Area under the curve of HDL levels in the four groups.

The levels of cholesterol in each rat group were tested every 2 weeks. As shown in Figure 1(A,B), there was no significant difference in cholesterol levels between LC (tAUC = 16.63) and LC + tocopheryl quinone (tAUC = 17.37) groups, while the HFCC (tAUC = 22.04) and HFCC + tocopheryl quinone (tAUC = 19.77) groups displayed higher cholesterol levels. Moreover, treatment with tocopheryl quinone decreased cholesterol levels in rats fed with HFCC. The levels of LDL in each rat group were also examined every 2 weeks. As shown in Figure 1(C,D), LDL levels were comparable in LC (tAUC = 1.745) and LC + tocopheryl quinone (tAUC = 1.795) groups, while the HFCC (tAUC = 2.961) and HFCC + tocopheryl quinone (tAUC = 2.359) groups displayed higher LDL levels. And the results of high-density lipoprotein (HDL) levels among the rat groups were similar to that of LDL (Figure 1(E,F)), with the LC (tAUC = 2.797) and LC + tocopheryl quinone (tAUC = 2.776) groups showing similar HDL levels, and the HFCC (tAUC = 3.303) and HFCC + tocopheryl quinone (tAUC = 3.035) groups displaying higher HDL levels. These results indicated that tocopheryl quinone could decrease blood lipid levels.

### Effects of HFCC and HFCC + tocopheryl quinone on oral glucose tolerance test (OGTT) in rats

**Figure 2.**
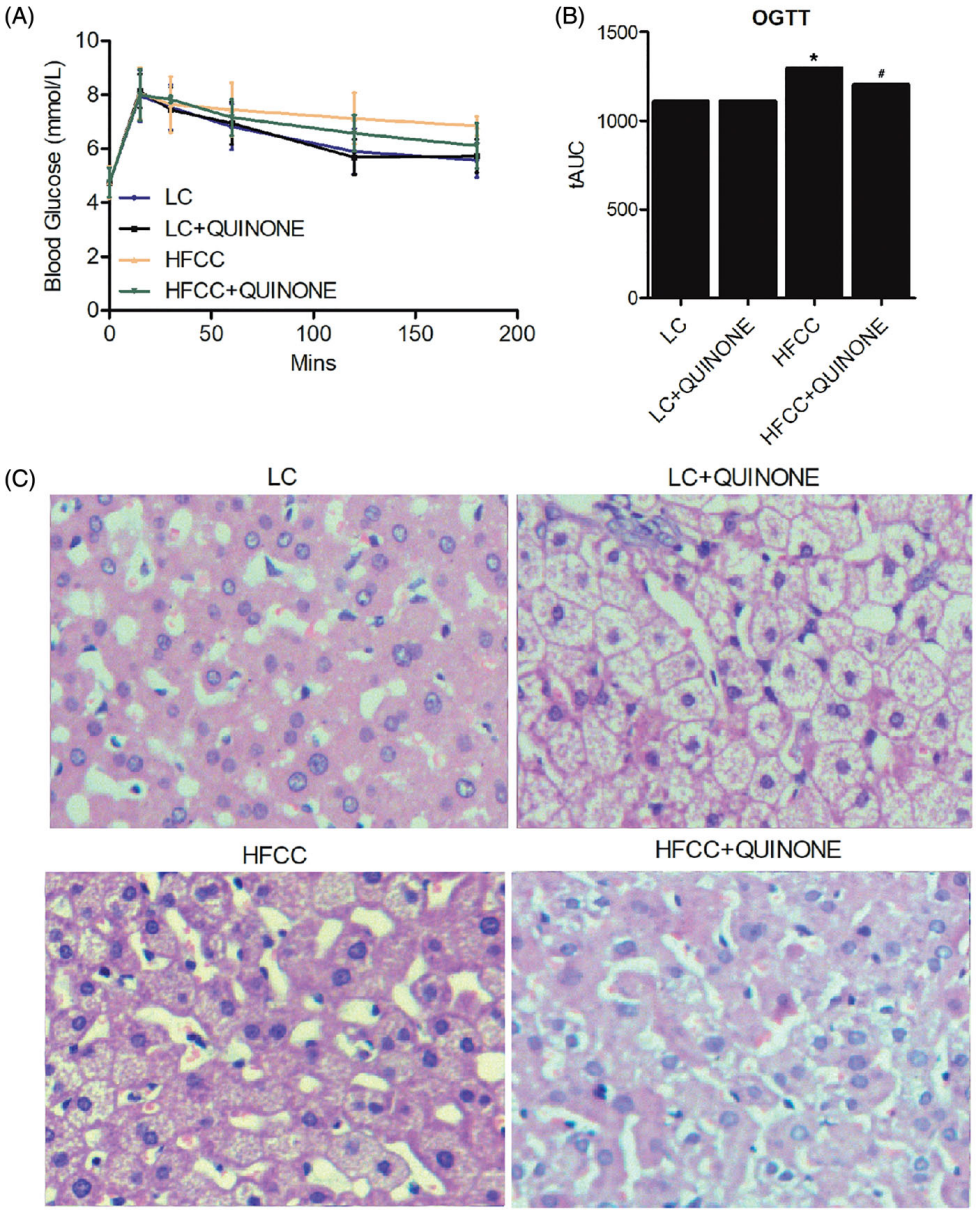
OGTT at different time points in LC group, LC + tocopheryl quinone group, HFCC group and HFCC + tocopheryl quinone group. The treatment with HFCC and tocopheryl quinone increased and decreased the levels of blood glucose, respectively (*n* = 3; *p value < 0.05 *vs.* LC group; ^#^*p* value < 0.05 *vs.* HFCC group). (A) Blood glucose levels at different time points in the four groups; (B) Area under the curve of blood glucose levels in the four groups. (C) H&E staining of liver tissue samples in the four groups.

The blood samples of the four groups were collected for an OGTT test. As shown in Figure 2(A,B), no difference was observed in blood glucose concentrations between LC (tAUC = 1152) and LC + tocopheryl quinone (tAUC = 1151) groups, while the HFCC (tAUC = 1296) and HFCC + tocopheryl quinone (tAUC = 1231) groups both showed higher blood glucose concentrations. Moreover, treatment with tocopheryl quinone increased blood glucose concentrations in rats fed with HFCC. These results indicated that tocopheryl quinone could maintain glucose homeostasis. The development of NASH in the animal models was further demonstrated by H&E staining of intestinal tissue samples (Figure 2(C)).

### Effects of HFCC and HFCC + tocopheryl tocopheryl quinone on the oxidative status of rats

**Figure 3.**
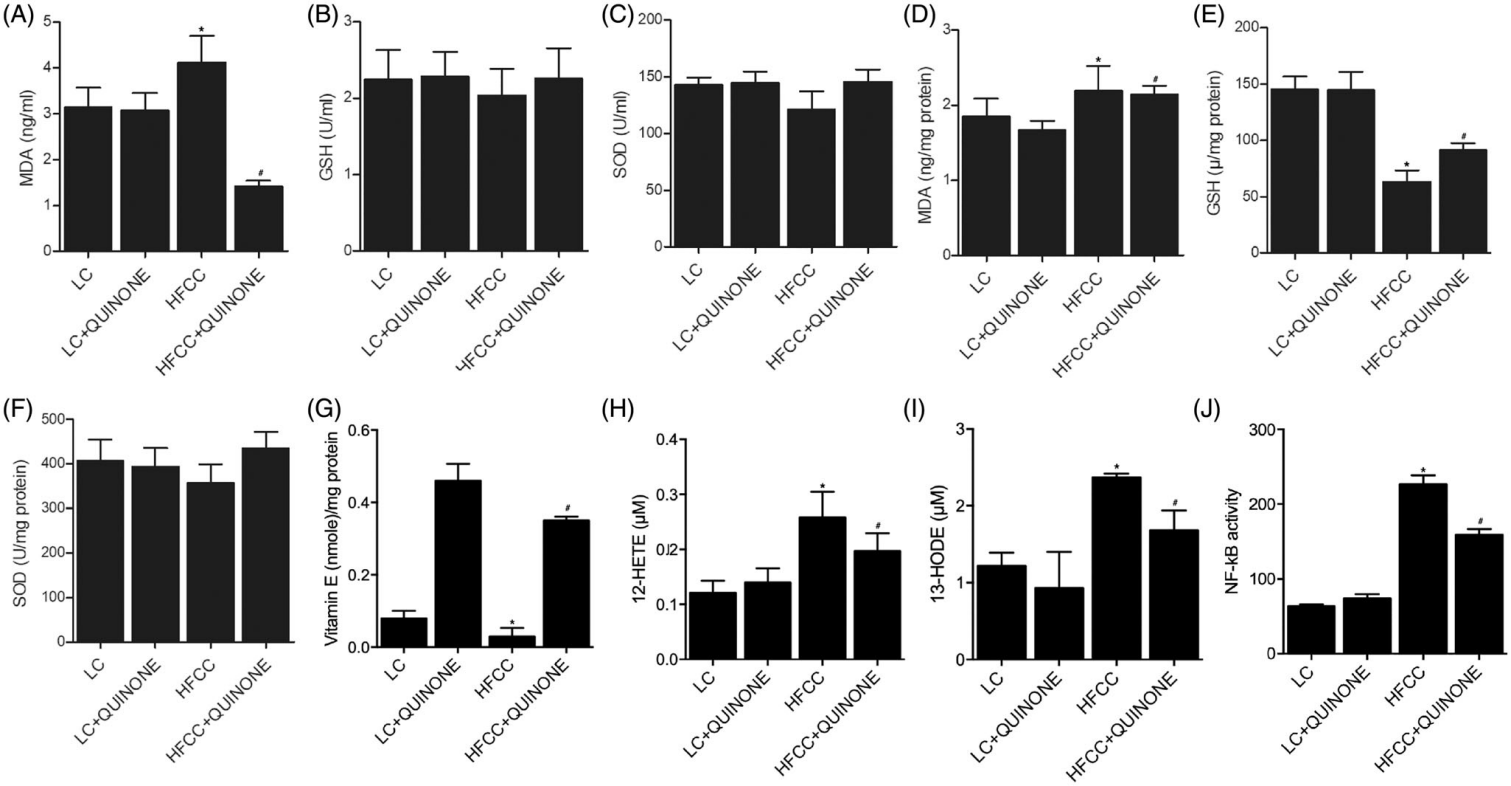
Expression levels of MDA, GSH, SOD, vitamin E, 12-HETE, 13-HODE and NF-κB in LC group, LC + tocopheryl quinone group, HFCC group and HFCC + tocopheryl quinone group. The treatment with HFCC and tocopheryl quinone increased and decreased the levels of redox indicators, respectively (*n* = 3; *p value < 0.05 *vs.* LC group; ^#^*p* value < 0.05 *vs.* HFCC group). (A) Serum MDA levels in the four groups; (B) Serum GSH levels in the four groups; (C) Serum SOD levels in the four groups; (D) MDA levels of intestinal tissues in the four groups; (E) GSH levels of intestinal tissues in the four groups; (F) SOD levels of intestinal tissues in the four groups; (G) Vitamin E levels of intestinal tissues in the four groups; (H) 12-HETE levels of intestinal tissues in the four groups; (I) 13-HODE levels of intestinal tissues in the four groups; (J) NF-κB activity of intestinal tissues in the four groups.

The levels of redox indicators (including MDA, GSH, SOD, vitamin E, 12-HETE and 13-HODE) in serum and intestinal tissues of the four groups were measured. As shown in Figure 3, the serum and tissue levels of MDA (Figure 3(A,D)), GSH (Figure 3(B,E)) and SOD (Figure 3(C,F)) were comparable in LC and LC + tocopheryl quinone groups, and the treatment with HFCC and tocopheryl quinone respectively increased and decreased the levels of redox indicators. Also, as indicated by Figure 3(G), the tissue expression of vitamin E was evidently increased by tocopheryl quinone treatment, while the tissue expression of 12-HETE (Figure 3(H)), 13-HODE (Figure 3(I)) and NF-κB (Figure 3(J)) was comparable between the LC and LC + tocopheryl quinone groups. The tocopheryl quinone treatment further decreased the up-regulated 12-HETE, 13-HODE and NF-κB in the HFCC group.

### Effect of HFCC and HFCC + tocopheryl quinone on the imbalance of intestinal flora

**Figure 4.**
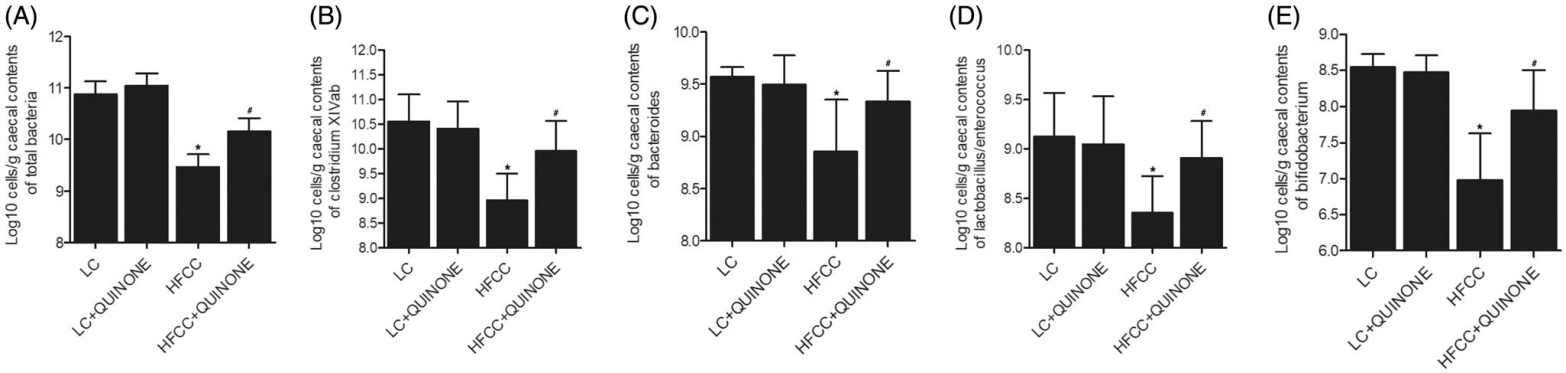
A 16S ribosomal ribonucleic acid–based analysis was used to test the intestinal flora in LC group, LC + tocopheryl quinone group, HFCC group and HFCC + tocopheryl quinone group (*n* = 3; *p value < 0.05 *vs.* LC group; ^#^*p* value < 0.05 *vs.* HFCC group). (A) Total number of bacteria in the four groups. The treatment with HFCC and tocopheryl quinone decreased and increased the total number of bacteria, respectively; (B) Number of *Clostridium* in the four groups. The treatment with HFCC and tocopheryl quinone decreased and increased the number of *Clostridium*, respectively; (C) Number of *Bacteroides* in the four groups. The treatment with HFCC and tocopheryl quinone decreased and increased the number of *Bacteroides*, respectively; (D) Number of *Lactobacilli* in the four groups. The treatment with HFCC and tocopheryl quinone decreased and increased the number of *Lactobacilli*, respectively; (E) Number of *Bifidobacteria* in the four groups. The treatment with HFCC and tocopheryl quinone decreased and increased the number of *Bifidobacteria*, respectively.

Subsequently, the profiles of intestinal flora in the four groups were assessed by a 16S ribosomal ribonucleic acid–based analysis. As shown in Figure 4, a significant decrease in the total number and diversity of bacteria was observed in the HFCC group. Additionally, the treatment with tocopheryl quinone substantially restored the numbers of bacteria, such as *Bacteroides*, *Bifidobacteria*, *Clostridium* and *Lactobacilli*.

### Effect of HFCC and HFCC + tocopheryl quinone on GLP-1 levels of intestinal tissues

**Figure 5.**
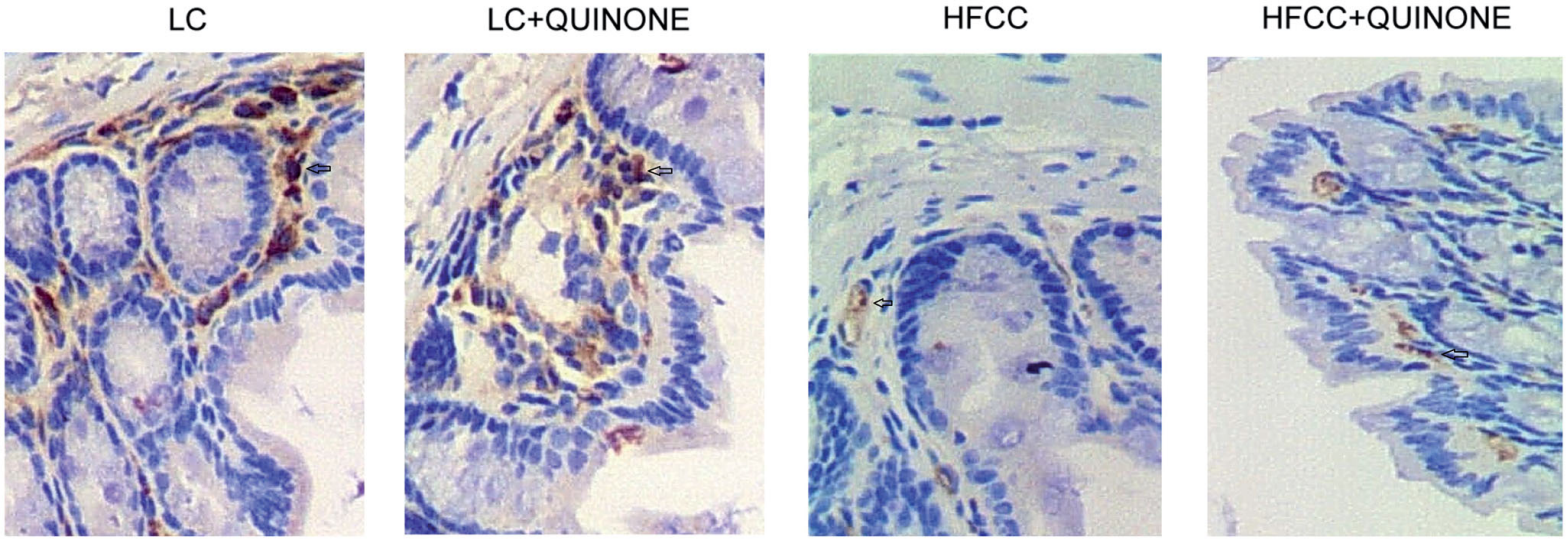
IHC was conducted to test the levels of GLP-1 in LC group, LC + tocopheryl quinone group, HFCC group and HFCC + tocopheryl quinone group. The treatment with HFCC and tocopheryl quinone increased and decreased the levels of GLP-1, respectively (*n* = 3, the brown as arrow-pointed denotes the expression of GLP-1).

**Figure 6.**
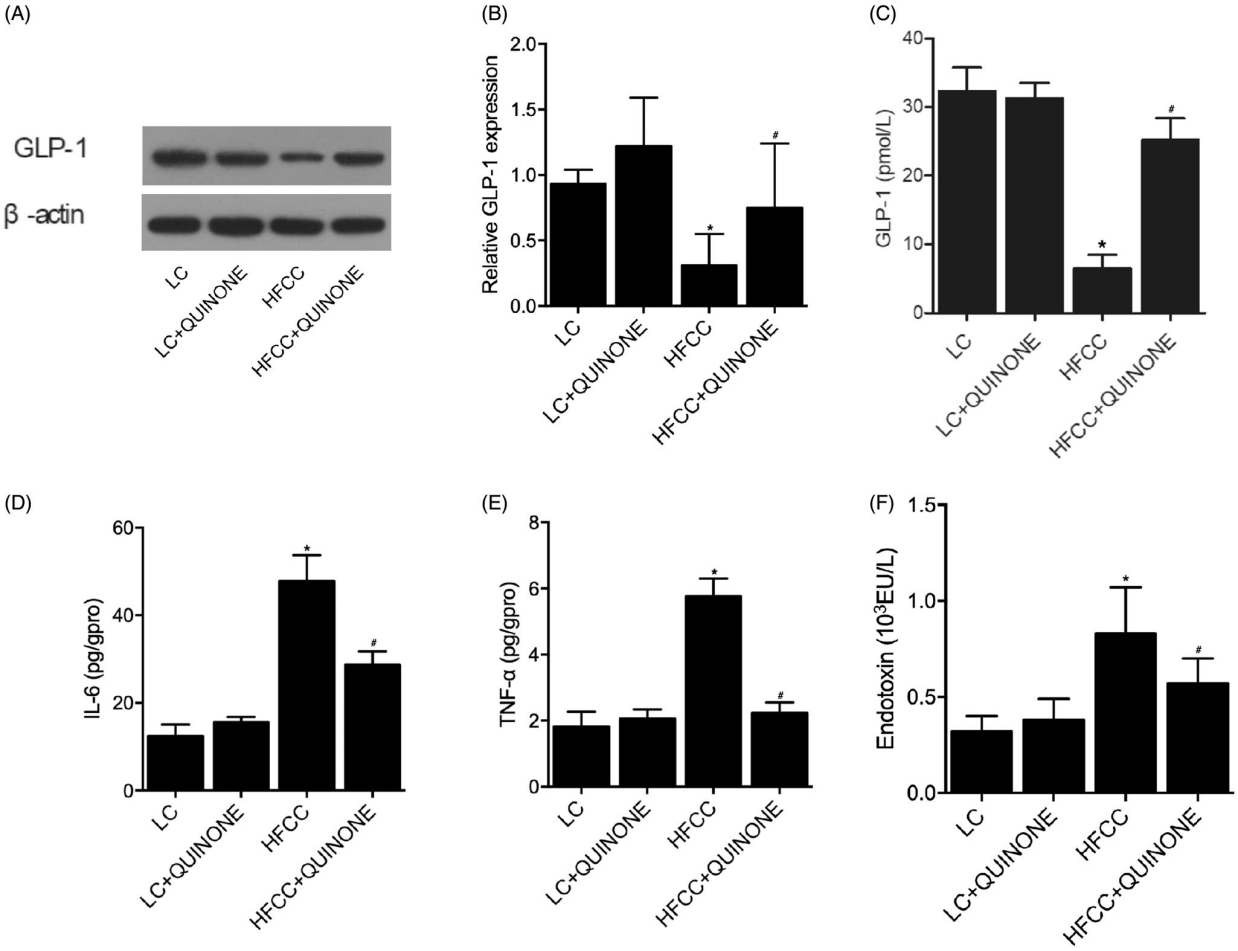
WB and ELISA were used to test the levels of GLP-1 in LC group, LC + tocopheryl quinone group, HFCC group and HFCC + tocopheryl quinone group (*n* = 3; *p value < 0.05 *vs.* LC group; ^#^*p* value < 0.05 *vs.* HFCC group). (A) Tissue levels of GLP-1 in the four groups were detected by WB. The treatment with tocopheryl quinone increased the expression of GLP-1 in the HFCC group; (B) Plasma levels of GLP-1 in the four groups were detected by ELISA. The treatment with tocopheryl quinone increased the expression of GLP-1 in the HFCC group; (C) Tissue activity of GLP-1 in the four groups was detected by ELISA. The treatment with tocopheryl quinone increased GLP-1 activity in the HFCC group; (D) Tissue IL-6 activity in the four groups was detected by ELISA. The treatment with tocopheryl quinone decreased IL-6 activity in the HFCC group; (E) Tissue TNF-α expression in the four groups was detected by ELISA. The treatment with tocopheryl quinone suppressed the expression of TNF-α in the HFCC group; (F) The expression of endotoxins in the HFCC group was reduced by the treatment with tocopheryl quinone.

The imbalance of intestinal flora could result in decreased GLP-1. Therefore, we tested the levels and activity of GLP-1 in intestinal tissues by IHC, Western blot and ELISA assays. As indicated by the results, the tissue (Figures 5 and 6(A)) and plasma levels of GLP-1 (Figure 6(B)) were comparable in LC and LC + tocopheryl quinone groups, while the treatment with HFCC and tocopheryl quinone increased the level of GLP-1. An ELISA assay was conducted to assess the activity of GLP-1, IL-6 and TNF-α in the four groups. As indicated by the results, the activity of GLP-1 (Figure 6(C)), IL-6 (Figure 6(D)), TNF-α (Figure 6(E)) and endotoxins (Figure 6(F)) was comparable in LC and LC + tocopheryl quinone groups, while the HFCC group showed decreased GLP-1 activity and increased IL-6 and TNF-α activity. Moreover, the treatment with tocopheryl quinone reversed the dysregulation in the activity of GLP-1, IL-6, TNF-α and endotoxins observed in the HFCC group.

### Effect of tocopheryl quinone on the growth of bacteria

**Figure 7.**
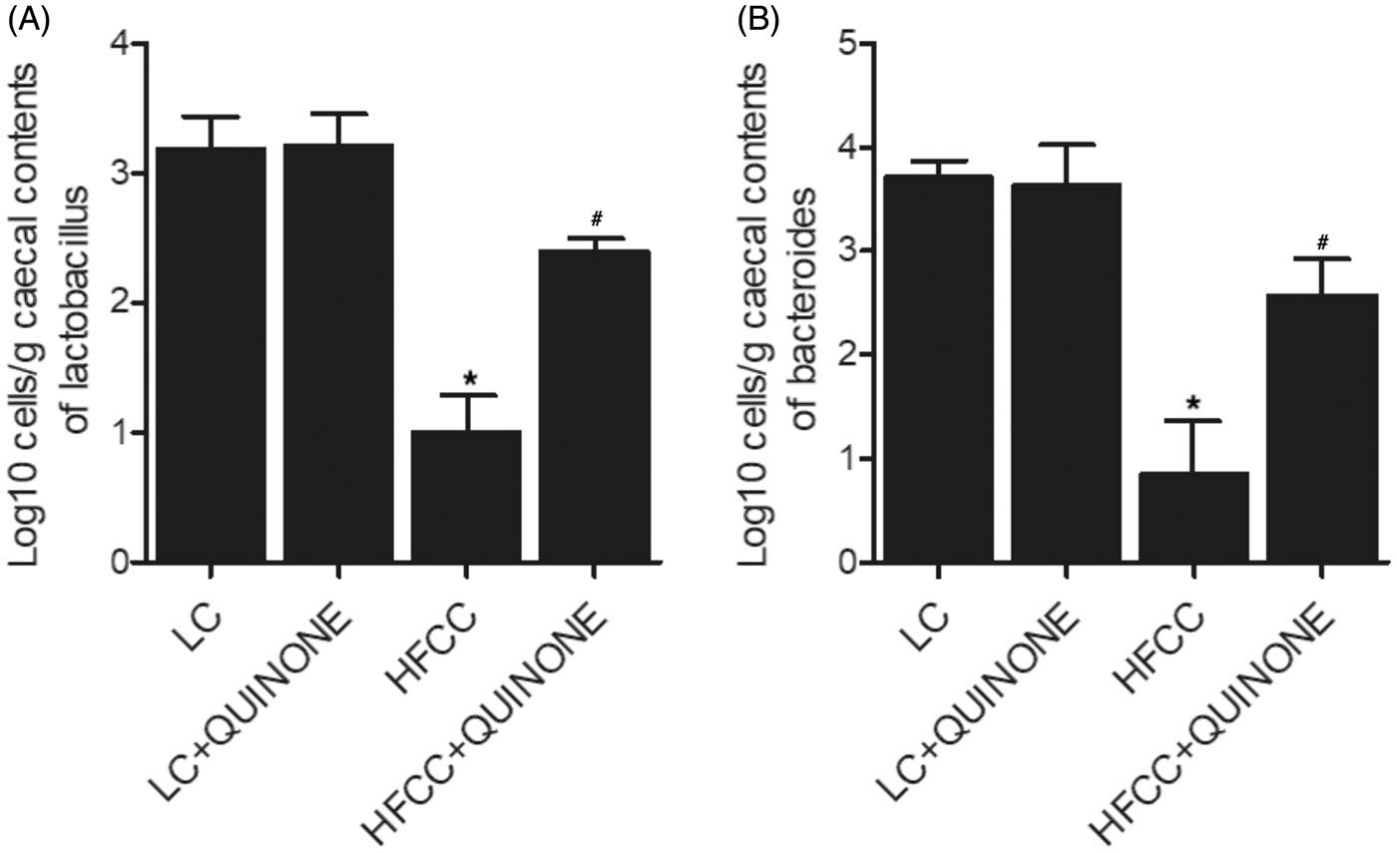
Effect of tocopheryl quinone on the growth of bacteria (*n* = 3; *p value < 0.05 *vs.* LC group; ^#^*p* value < 0.05 *vs.* HFCC group). (A) Treatment with tocopheryl quinone significantly promoted the growth of *Lactobacilli*; (B) Treatment with tocopheryl quinone significantly promoted the growth of *Bacteroides*.

Subsequently, the effects of tocopheryl quinone on the growth of bacteria were inspected *in vitro*. As shown in Figure 7, the treatment with tocopheryl quinone significantly promoted the growth of both *Lactobacilli* (Figure 7(A)) and *Bacteroides* (Figure 7(B)) *in vitro*.

## Discussion

In this study, we set up the animal model of NASH and treated the animals with tocopheryl quinone, finding that the levels of cholesterol, LDL and HDL were comparable in LC and LC + tocopheryl quinone groups, while the HFCC and HFCC + tocopheryl quinone groups displayed higher levels of cholesterol, LDL and HDL. In addition, an OGTT was performed. No difference was observed in blood glucose concentrations between LC and LC + tocopheryl quinone groups, while the treatment with HFCC and tocopheryl quinone increased and decreased blood glucose concentrations, respectively. The results indicated that the lipid-reducing effect of tocopheryl quinone is only effective in the animals with hyperlipidaemia or hyperglycosemia but not in normal ones.

Tocopherol, a phenolic antioxidant, is one of the parent congeners in the vitamin E family. The oxidation reactions during tocopheryl quinone electrophiles lead to the formation of TQ, the biologically active component of tocopherol (Cornwell and Ma 2007). In addition, the therapeutic effect of TG was found to be mediated by its ability to modulate the intestinal flora balance. The important effects of microbiota on the progression of NASH have been demonstrated in NASH rats fed with a high-fat diet. It was found that the transplantation of faecal microbiota collected from donor rats fed with a standard chow attenuated the severity of NASH by decreasing the level of liver adiposity and by decreasing the production of inflammatory cytokines (Zhou et al. 2017). Similar studies in rodents demonstrated that the treatment with probiotics slows the development of NASH and the progression of liver disorders (Kim et al. 2017; Xue et al. 2017). In addition, the level of microbial DNA in the blood samples collected from obesity patients is increased along with a decreased level of microbial biodiversity upon the development of liver fibrosis (Lelouvier et al. 2016). As a result, a unique microbial profile may indicate the onset and susceptibility to NASH. Moreover, it is well known that the imbalance in gut microbiota can lead to diabetes (Munoz-Garach et al. 2016). Interestingly, in children patients of diabetes, the levels of *Bacteroides*, *Actinobacteria*, and *Firmicutesto Bacteroidetes* are significantly reduced (Murri et al. 2013). Similarly, Roesch et al. (2009) showed that the population of *Ruminococcaceae* and *Clostridiaceae* was more enriched in faeces collected from HFCC rats.

Tocopherol quinone was demonstrated to play a significant antioxidant role in a wide range of biological systems. Initially deemed as a predictor of oxidant damages, α-TQ is highly enriched in body fluids of infants (Vatassery 1989; Jain et al. 1996). In addition, α-TQ is typically used to treat oxidative stress in elderly patients with NIDDM. However, it is worth noting that the level of plasma α-TQ in these elderly NIDDM patients is apparently reduced. In line with this, we found that, this study, the levels of redox indicators (including MDA, GSH, SOD and vitamin E) in the serum and intestinal tissue samples were measured. The serum levels of these redox indicators were comparable in LC and LC + tocopheryl quinone groups, while the treatment with HFCC and tocopheryl quinone increased and decreased the levels of redox indicators, respectively.

As a peptide containing 30 amino acids, GLP-1 plays an essential role in inducing the uptake of blood glucose by the cells in body tissues (Bell et al. 1983; Schmidt et al. 1985; Mojsov et al. 1986; Orskov et al. 1986). In addition, GLP-1 can act as an incretin to stimulate the postprandial uptake and metabolism of blood glucose. As a result, GLP-1 has been widely used in clinical applications to treat patients with type II DM (Mojsov et al. 1986; Gros et al. 1993). In this study, IHC and Western blot revealed that GLP-1 levels were comparable in LC and LC + tocopheryl quinone groups, while the treatment with HFCC and tocopheryl quinone increased and decreased the levels of GLP-1, respectively. The ELISA assay further confirmed that the treatment with HFCC and tocopheryl quinone increased and decreased the activity of GLP-1, respectively. A previous study has demonstrated that incretin-related drugs can act as DPP4 inhibitors and agonists of GLP-1 receptors. However, the effectiveness of DPP4 inhibitors in the treatment of diabetic patients with NASH/NAFLD has been controversial. In several clinical studies with a small sample size, the effectiveness of liraglutide, an agonist of GLP-1 receptors, was obvious in Japanese patients with NASH (Eguchi et al. 2015; Sumida et al. 2017). Therefore, semaglutide and dulaglutide have been tested as two agonists of GLP-1 receptors in diabetic patients with NASH, and the results of these clinical trials are promising (Katsiki et al. 2017; Sumida et al. 2017). Nevertheless, based on the guidance of the American Association for the Study of Liver Diseases (AASLD), the use of GLP-1 receptor agonists in the treatment of diabetic NAFLD/NASH patients has not been fully verified due to the lack of sufficient evidence supporting their efficacy (Chalasani et al. 2018). In addition, diabetic NASH patients may benefit more from novel treatments specifically targeting diabetes, including SGLT2 inhibitors. For example, as a type of SGLT2 inhibitors, pemafibrate has shown promising efficacy in the treatment of patients suffering from both dyslipidemia and NASH (Sourianarayanane et al. 2013). It was also demonstrated in diabetic rats that the treatment with GLP‐1 can reduce their serum levels of MDA and glucose as well as their expression of IL‐1β and IL‐6, along with an effect to elevate the production of catalase, insulin and SOD (Yuan et al. 2018).

## Conclusions

The findings of this study confirmed the effect of α-tocopheryl quinone on GLP-1 activity and demonstrated the therapeutic effect of tocopheryl quinone in the treatment of NASH-associated DM by restoring the balance of intestinal flora.

## Data Availability

The data sets generated during and/or analysed during this study are available from the corresponding author on reasonable request.
